# An Account of Diseases in an Eastern District of London, from May 20 to June 20, 1806

**Published:** 1806-07

**Authors:** 


### *An Account of Diseases in an Eastern District of London, from May* 20 *to June* 20, 1806.

| | |
|---|---|
| ACUTE DISEASES. | |
| Scarlatina Anginosa - | 3 |
| Rubeola - - - | 2 |
| Ophthalmia - - - | 3 |
| Rheumatismus Acutus | 1 |
| CHRONIC DISEASES. | |
| Tussis - - - | 7 |
| Dyspnœa - - - | 6 |
| Tussis cum Dyspnœa - | 5 |
| Phthisis Pulmonalis - | 1 |
| Asthma | 1 |
| Anasarca - - - | 2 |
| Ascites - - - | 3 |
| Epistaxis - - | 1 |
| Cephalalgia - - | 6 |
| Cardialgia - - | 4 |
| Fluor Albus - - | 10 |
| Amenorrhœa - - | 4 |
| Menorrhagia - - | 5 |
| Hæmorrhois - - | 6 |
| Hysteria - - | 2 |
| Rheumatismus Chronicus | 16 |
| PUERPERAL DISEASES. | |
| Ephemera - - | 7 |
| Rhagas Papillæ | 3 |
| INFANTILE DISEASES. | |
| Tinea - - - | 2 |
| Herpes - - - | 5 |
| Erysipelas Infantilis - | 1 |

The change which has taken place in the temperature of the air during the last few weeks, has produced a considerable alteration in the state of diseases. The number of coughs and catarrhs, and of other pneumonic affections, is considerably lessened; and the symptoms of those which which have not entirely subsided, have appeared in a milder form.

Scarlatina anginosa has occurred in several instances; this, as is usually the case, particularly affected young people. It began with slight shiverings, which were succeeded by some uneasiness in the throat. The tonsils swelled, and were inflamed, and, of course, deglutition became difficult. An eruption appeared, which continued for a few days, and then went off in the usual manner, with a desquamation.

In each of the cases referred to, an anasarcous swelling came on after other symptoms had disappeared; but this gradually subsided by the use of a few laxative medicines.

